# Ferroptosis of epithelial ovarian cancer: genetic determinants and therapeutic potential

**DOI:** 10.18632/oncotarget.27749

**Published:** 2020-09-29

**Authors:** Chao-Chieh Lin, Jen-Tsan Chi

**Affiliations:** ^1^Department of Molecular Genetics and Microbiology, Duke University School of Medicine, Durham, NC 27710, USA; ^2^Center for Genomic and Computational Biology, Duke University School of Medicine, Durham, NC 27710, USA

**Keywords:** ferroptosis, cystine, ovarian cancer, GPX4, NADPH oxidase

## Abstract

Epithelial ovarian cancer (OVCA) is the most lethal gynecologic cancer. Current treatment for OVCA involves surgical debulking of the tumors followed by combination chemotherapies. While most patients achieve complete remission, many OVCA will recur and develop chemo-resistance. Whereas recurrent OVCA may be treated by angiogenesis inhibitors, PARP inhibitors, or immunotherapies, the clinical outcomes of recurrence OVCA are still unsatisfactory. One new promising anti-tumor strategy is ferroptosis, a novel form of regulated cell death featured by lipid peroxidation. In this review, we have summarized several recent studies on the ferroptosis of OVCA. Also, we summarize our current understanding of various genetic determinants of ferroptosis and their underlying mechanisms in OVCA. Furthermore, ferroptosis can be combined with other standard cancer therapeutics, which has shown synergistic effects. Therefore, such a combination of therapeutics could lead to new therapeutic strategies to improve the response rate and overcome resistance. By understanding the genetic determinants and underlying mechanisms, ferroptosis may have significant therapeutic potential to improve the clinical outcome of women with OVCA.

## CURRENT THERAPEUTIC STRATEGIES FOR ADVANCED OVARIAN CANCER

Epithelial ovarian cancer (OVCA) originates from a layer of cells covering the surface of the ovaries or fallopian tubes and accounts for ~90% of the primary ovarian tumors [1]. Throughout the world, OVCA is the most lethal gynecologic cancer, with 46% survival five years after diagnosis [2]. NCI Seer data (https://seer.cancer.gov/) predict that approximately 21,750 American women will be diagnosed with OVCA in 2020, which will lead to the death of 13,940 American women. The diagnosis of OVCA is challenging due to the vague and non-specific symptoms at the initial stage. Thus, OVCA is often misdiagnosed as other common ailments. Moreover, due to the deficiency of early-stage OVCA screening strategies, the correct diagnosis of OVCA usually occurs at advanced stages, resulting in poor prognosis and low survival rate [3, 4]. For most low-grade OVCA confined in ovaries and pelvis, a debulking surgery is curative. For high-grade OVCA, standard therapy involves surgical debulking of the tumors followed by combination chemotherapies with carboplatin and paclitaxel [5]. Most patients initially respond favorably to this combined treatment and achieve remission [6]. However, in many cases, tumors will eventually recur, and recurrent tumors will become resistant to chemotherapies, which were effective for primary tumors. Therefore, angiogenesis inhibitors, PARP inhibitors, and immunotherapies are employed to treat recurrent OVCA [7, 8].

Angiogenesis inhibitors aim to inhibit the growth of new blood vessels in tumors by blocking the vascular endothelial growth factor (VEGF), VEGF receptors, or its downstream signaling pathway [9]. In most solid tumors, including OVCA, the uncontrolled tumor growth, combined with inadequate blood perfusion, leads to low tumor pO2, tumor hypoxia, and other tumor microenvironmental stresses [10–13]. Hypoxia triggers the HIF-mediated hypoxia gene expression program that leads to the invasion, migration, and metastasis of tumor cells [14–16]. Tumor hypoxia also induces abnormal angiogenesis, creates dysregulated blood vessel networks defective in drug delivery and contributes to chemo-resistance [14–16]. Therefore, angiogenesis inhibitors, such as bevacizumab, may normalize tumor blood vessels, mitigate tumor hypoxia, and restore response to chemotherapeutics [17].

Poly (ADP-ribose) polymerases (PARPs) are a family of proteins that catalyze the transfer of ADP-ribose to target proteins (poly ADP-ribosylation). PARPs mediate many biological processes, including the repair of single-strand break (SSBs) through base excision repair [18]. PARP inhibitors, such as Olaparib and Rucaparib, have emerged as effective treatments for a subset of OVCA bearing mutations in *BRCA1* and *BRCA2* [18]. Both BRCA1 and BRCA2 proteins promote homology-directed repair (HDR) of DNA double-strand break (DSB) [19]. Therefore, *BRCA1* and *BRCA2* mutations lead to defects in DNA DSB repair, rendering these *BRCA1* and *BRCA2* mutated cells rely on a PARP-mediated DNA repair pathway. Hence, they are highly sensitive to the death caused by PARP inhibitors [20, 21]. In contrast, healthy cells with intact BRCA1/BRCA2 are not susceptible to PARP inhibitors, creating the synthetic lethal relationship and significant therapeutic window [20, 21]. Olaparib was initially approved for maintenance for BRCA-mutated recurrent OVCA [18]. Recently, the FDA expanded the approval of Olaparib and bevacizumab as the front-line treatment for women with advanced ovarian cancer [22].

Immune checkpoint blockade is a powerful new therapeutic option for many cancers [23]. The most common immune checkpoint blockage refers to blocking immune inhibitory receptors (CTLA4, PD1 on T cells, or PDL1 on tumor cells and tumor-infiltrating immune cells) using antagonistic antibodies. Programmed death 1 (PD1) and its ligands PDL1 and PDL2 play a key role in dampening T cell responses in the tumor [24–26]. Blocking the PD1/PDL1 inhibitory axis allow the CD8+ CTL to attack tumor cells, leading to a sustained anti-tumor response. However, the clinical responses of advanced OVCA to immunotherapy are unsatisfactory, with response seen in only 10–25% patients [27–31]. Therefore, there are increasing interests in combining other novel therapeutic approaches with the immune checkpoint blockade to improve response rate and efficacy.

## FERROPTOSIS–A NOVEL FORM OF REGULATED CELL DEATH WITH SIGNIFICANT THERAPEUTIC POTENTIAL

Even with all these advancements, clinical outcomes of advanced OVCA are still unsatisfactory [7]. Therefore, new therapeutic options are urgently needed. One new strategy to eliminate tumor cells is to identify and target their metabolic Achilles’ heel and specific nutrient preference [32]. Cystine deprivation of cancer cells with specific cellular origins and somatic mutations triggers ferroptosis, a novel form of regulated cell death characterized by lipid peroxidation [33, 34]. Ferroptosis was first uncovered during the investigation of the death mechanisms induced by erastin, an agent that was selected based on its ability to selectively eradicate RAS-mutated cancer cells [33]. Since then, significant progress has been made in understanding the biological processes and genetic determinants of ferroptosis, as summarized in some excellent reviews [35–38]. Here we review some key players relevant to the ferroptosis of OVCA.

### GPX4 and FSP1 mediate two ferroptosis protection pathways

There are two known ferroptosis protection mechanisms mediated by glutathione peroxidase 4 (GPX4) and ferroptosis suppressor protein 1 (FSP1). Both proteins neutralize ROS and prevent lipid peroxidation. GPX4 is a phospholipid hydroperoxidase that protects cells against membrane lipid peroxidation using glutathione (GSH) as its cofactor. Therefore, ferroptosis can be triggered by either the depletion of GSH or direct inhibition of GPX4. RSL3 and several other ferroptosis-inducing agents (FINs) [39] induce ferroptosis by blocking the function of GPX4 downstream of the NADPH-GSH that supply the cofactors for GPX4.

Many ferroptosis-inducing agents work by the depletion of GSH or cysteine. For example, erastin is an xCT inhibitor that induces ferroptosis by preventing cystine import and depleting GSH. Similarly, the cystine deprivation also leads to GSH depletion and death in a subset of cystine-addicted cancer cells [40–42]. In contrast, enhanced GSH synthesis upon the activation of NRF2 by various mechanisms would protect cells from ferroptosis [43, 44].

Several pathways can also compensate for the cystine deprivation and rescue ferroptosis. Upon cystine deprivation or xCT inhibitors, the cysteine can be generated by the transsulfuration pathway to prevent ferroptosis. For example, a forward genetic screen revealed that the removal of cysteinyl-tRNA synthetase (CARS) protected ferroptosis [45]. This protection occurred through the induction of the transsulfuration pathway to replenish cysteine [45]. Furthermore, the addition of coenzyme A (CoA), from the *de novo* CoA synthesis pathway [46], also replenishes cysteine and rescued ferroptosis [47]. In addition, pharmacogenomic analyses identified NAPDH as a robust determinant of ferroptosis [48], probably by regenerating GSH. Consistently, we have found that MESH1, the metazoa homolog of SpoT, is the first cytosolic NADPH phosphatase [49] whose induction is responsible for the NAPDH depletion during ferroptosis [50].

FSP1 and Coenzyme Q_10_ (CoQ_10_) axis have been identified as a new ferroptosis protection mechanism [51, 52]. FSP1 is an NADH-dependent CoQ_10_ oxidoreductase that reduces CoQ_10_. When FSP1 is myristoylated, it moves to the plasma membrane to limit lipid peroxidation and suppress ferroptosis. Therefore, the removal of FSP1 also leads to lipid peroxidation, membrane damage, and ferroptosis.

### Promotion of ferroptosis by NOXs and iron

During ferroptosis, the oxidative radicals are generated by NOXs (nicotinamide adenine dinucleotide phosphate (NADPH) oxidases), a family of oxidases that use NADPH as cofactors. Therefore, NOX inhibitors consistently inhibit ferroptosis. Interestingly, each member of NOXs expresses differently in a tissue-specific manner [53]. Therefore, the specific members of the NOXs mediate ferroptosis may vary in distinct cell and tissue types.

Iron metabolism and labile iron pools are also critical for ferroptosis [33]. “Ferroptosis” indicates that iron is indispensable. Iron is postulated to drive the Fenton reaction that amplifies the free oxidative radicals, generated by NOXs and other sources, to trigger ferroptosis [54]. Therefore, iron chelator blocks ferroptosis by limiting cellular iron levels. For example, enhanced ferroptosis susceptibility is noted in erythrocyte-ingested macrophages [55] and hepatocytes in patients with hemochromatosis [56].

Similarly, NRF2 activation also limits ferroptosis by inducing the transcription of Ferritin Heavy Chain 1 (*FTH1*, involved in iron storage) to reduce labile iron [57]. Chen *et al.* found that the serine/threonine kinase ATM involved in the DNA damage pathway also regulated ferroptosis. Inhibition of ATM by genetic and chemical means prevents ferroptosis by reducing cellular iron through the induction of both iron storage (*FTH1*, *FTL* – Ferritin Heavy, and Light Chain), and export (*FPN1*-Ferroportin) [58].

## FERROPTOSIS SUSCEPTIBILITY OF OVARIAN CANCER CELLS AND RELEVANT GENETIC DETERMINANTS

**Figure 1:**
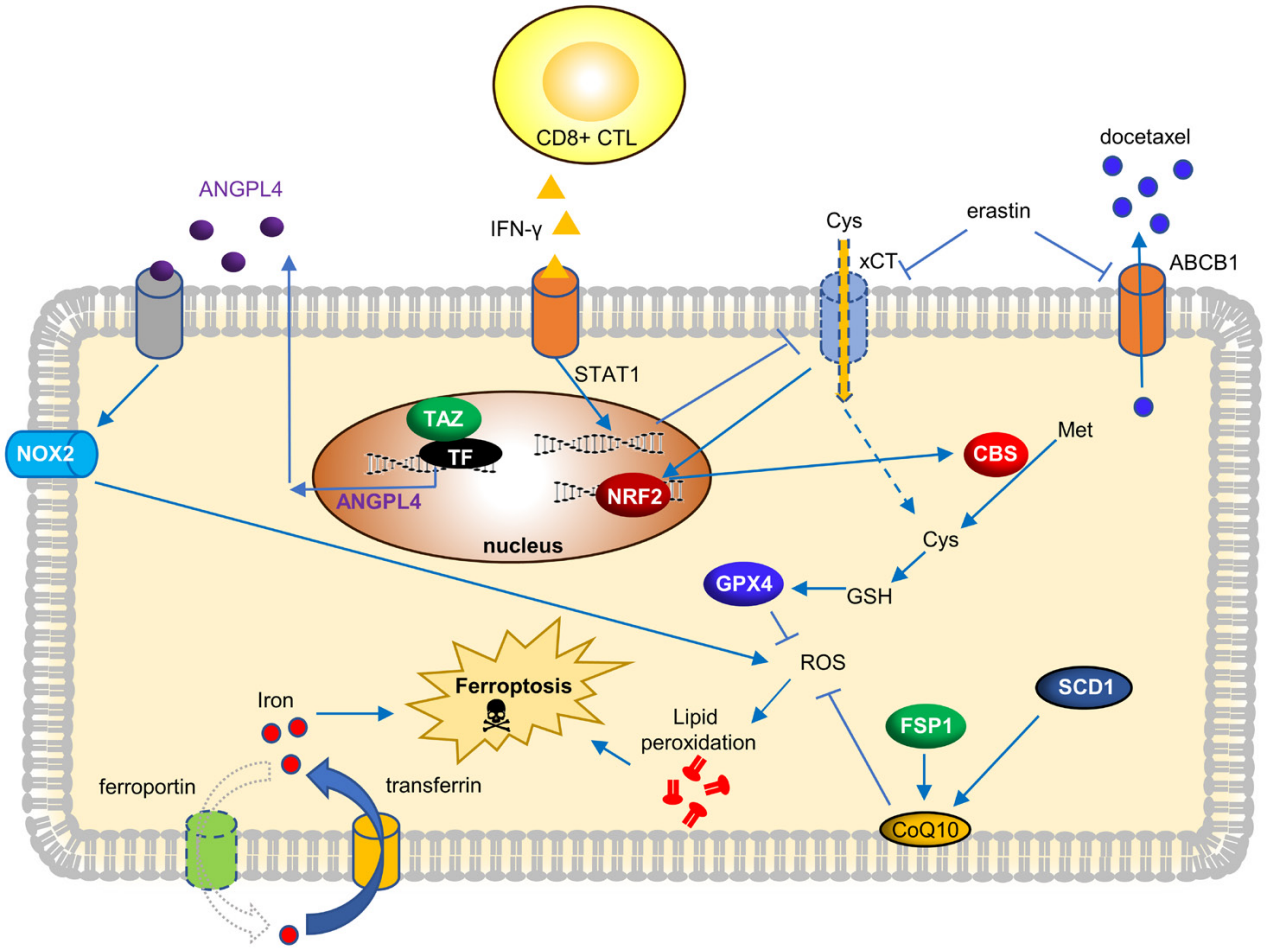
Current genetic determinants and its molecular mechanisms to trigger ferroptosis in OVCA.

Inducing ferroptosis has been shown to have potent anti-tumor potential for many tumor types [35–37]. However, relatively little is known about the determinants and therapeutic potential of ferroptosis in OVCA. Several recent studies have investigated the genetic determinants of ferroptosis and demonstrated the potential role in OVCA therapy. We summarize these studies and their findings in Figure 1. Torti group first described that the high-grade OVCA has a lower level of the iron exporter (ferroportin) and a higher level of the iron importer (transferrin receptor), resulting in the accumulation of intracellular labile iron. High intracellular labile iron enhances the invasion and metastasis of OVCA by inducing matrix metalloproteases and interleukin 6 [59]. Since the iron chelators can eradicate these OVCA, they termed such observations “iron addiction” [59]. As expected, such high intracellular iron also promotes the ferroptosis of these OVCA [59]. In the follow-up studies, the Torti group elucidated the role of stearoyl-CoA desaturase (SCD1) in ferroptosis. SCD1 catalyzes the rate-limiting step in the monounsaturated fatty acid synthesis. Inhibition of SCD1 depletes CoQ10, an endogenous membrane antioxidant used by FSP1 to protect cells from ferroptosis [60] (Figure 1).

The altered metabolism of cancer cells may render specific nutrients indispensable. Such nutrient addiction can be identified in a nutrigenetic screen by dropping off individual nutrient and analyze its transcriptome responses and cell viability [61]. Such a nutrigenetic approach revealed glutamine [62] and cystine addiction of renal cell carcinoma [40], breast cancer [41] and non-small cell lung cancers (NSCLC) [42]. When a similar nutrigenetic screen was used to analyze a panel of serous and clear-cell type OVCA cells, these OVCA cells were highly sensitive to cystine-deprived death [63]. Interestingly, cell density seemed to be a critical factor. As the Hippo effectors YAP/TAZ are the sensors of cell density [64], we identified that TAZ regulated ferroptosis in OVCA by affecting the expression of ANGPTL4 and NOX2 [63]. Therefore, ferroptosis-inducing agents may have significant therapeutic potential for OVCA with activated TAZ [63].

The immunotherapy depends on the ability of the CD8+ CTL to eradicate target tumor cells. Li’s group recently showed that CD8+ CTL and γ-interferon (IFN-γ) killed ID8, a murine OVCA cell, through the ferroptosis mechanism [65]. IFN-γ suppressed the cystine import by repressing the expression of *SLC3A2* and *SLC7A11*, both subunits of the xCT that mediate the cystine import (Figure 1). Importantly, ferroptosis-inducing agents can enhance the efficacy of immunotherapy. This landmark study draws the unexpected connection between ferroptosis and immunotherapy.

One major clinical challenge of OVCA is the chemo-resistance of recurrent OVCA [7, 8]. One mechanism of docetaxel resistance is the overexpression ATP Binding Cassette Subfamily B Member 1 (*ABCB1*), which pumps out the docetaxel [66]. A recent study on the docetaxel-resistant OVCA [67] has shown that erastin mitigates the overexpression of *ABCB1*. Thus, when erastin was combined with docetaxel, erastin significantly increased the intracellular level of docetaxel. Therefore, erastin reverses the ABCB1-mediated chemo-resistance in OVCA, showing the therapeutic value of combining erastin and docetaxel [67] (Figure 1).

Another interesting paper focuses on ferroptosis resistance in OVCA induced by long-term erastin exposure [67]. Prolonged erastin eventually leads to ferroptosis resistance by activation of the transsulfuration pathway. Ferroptosis resistance is caused by the NRF2-mediated upregulation of cystathionine β-synthase (CBS) and transsulfuration. Therefore, genetically repression of NRF2 enhanced ferroptosis susceptibility of these ferroptosis-resistant cells, consistent with the anti-ferroptosis role of NRF2 [43, 44, 68].

## THERAPEUTIC IMPLICATION AND FUTURE DIRECTION

These studies have provided compelling evidence that OVCA is highly sensitive to ferroptosis. However, much remained unknown about the genetic determinants of ferroptosis in OVCA to enable the selection of ovarian tumors, which may best respond to ferroptosis-inducing therapies. First, OVCA is an extremely heterogeneous disease based on the histopathology, somatic mutations, cellular origins, and various clinical parameters. For example, OVCA is classified into different histological subtypes, including serous, mucinous, endometrioid, clear cell, transitional cell, carcinosarcoma, mixed epithelial tumor, and undifferentiated carcinoma [69]. It is not clear whether these histological types guide the use of ferroptosis for OVCA. While most of the current studies of ferroptosis focus on the serous OVCA, future efforts will expand the investigations to other histological types. It is interesting to note that clear-cell type OVCA, characterized by the clear cytoplasm due to lipid and glycogen accumulation, is highly addicted to cystine and sensitive to the GPX4-removal ferroptosis [70]. However, the ferroptosis phenotypes of other OVCA remain largely unknown.

Another critical source of OVCA heterogeneity is the somatic mutations. TGCA analysis of OVCA has revealed the landscapes of somatic mutations [71]. As expected, *TP53* was found to be mutated in > 90% of tumors. The next most common mutations are in *BRCA1* or *BRCA2* in 11–12% of OVCA. Other statistically recurrently mutated genes include *RB1*, *NF1*, *FAT3, CSMD3, GABRA6,* and *CDK12* [71]. TP53 is one of the most important tumor suppressor genes, and different mutations of TP53 have been reported to either promote or limit ferroptosis in a highly context-dependent manner [72–75]. Therefore, it will be fascinating to elucidate further whether and how p53 mutations affect the ferroptosis in OVCA. Other than p53, retinoblastoma protein (*RB1*) also limited the sorafenib-induced ferroptosis [76]. Therefore, these somatic mutations may alter the metabolic states of the OVCA to enhance or limit ferroptosis sensitivity. However, much remains unknown about how to incorporate these histological subtypes and somatic mutations into reliable and robust predictors of ferroptosis sensitivity of OVCA.

Even many studies have identified genetic determinants of ferroptosis in other cancer types, it will still be important to validate and identify the specific determinants in OVCA. For example, the Hippo pathway has been shown to regulate ferroptosis in multiple tumor cell types [63, 77–80]. However, different Hippo effectors are employed in different cancer cells. In breast cancer and mesothelioma, YAP regulates ferroptosis in response to cellular contacts [79]. In contrast, in renal and ovarian cancer, TAZ is the relevant Hippo effector [63, 78] due to the predominant expression pattern. Similarly, while NOXs are essential for ferroptosis, distinct NOX members execute ferroptosis in different tumors. In OVCA, NOX2 was highly expressed to mediate ferroptosis [63]. In contrast, the ferroptosis of renal cell carcinoma is mediated by renal-specific NOX4 [78]. Therefore, Identifying the particular genetic determinants and relevant mediators of ferroptosis in OVCA may help to predict the response to ferroptosis-inducing therapies and potential resistant mechanisms.

Employing ferroptosis in combination therapeutics may have the opportunities to enhance the efficacy of existing therapeutic approaches. Ferroptosis is found to enhance the efficacy of immunotherapies [65], chemotherapies [81], and ionization radiations [82–84]. DNA damage and ATM/ATR activation have been found to promote ferroptosis [58]. PARP inhibitors may also trigger DNA damage, ATM/ATR activation [85], thus sensitizing OVCA to ferroptosis. Therefore, future efforts on optimizing the best strategies combining ferroptosis with standard cancer therapeutics would greatly improve outcomes and survival of patients with advanced OVCA.

While xCT inhibitors and cystine deprivation are established means to induce ferroptosis *in vitro*, it is not clear how best to induce ferroptosis *in vivo* for therapeutic purposes. Recently, imidazole ketone erastin (IKE) has been developed for *in vivo* application because of its potency, solubulity and metabolic stability [86]. Another promising agent with significant translational potential is the engineered human cyst(e)inase modified from CBS [87]. Cyst(e)inase suppresses tumor growth in multiple syngeneic and xenograft tumor models without apparent weight loss or other adverse effects. Cyst(e)inase can also synergize with immunotherapy [65] and is effective in pancreatic cancers [47]. Therefore, these reagents will be further optimized for the future clinical application of triggering ferroptosis to improve the outcomes of women with advanced OVCA. Studies have demonstrated a different angle on targeting anti-ferroptosis components, GPX4, or FSP1. However, it remains unknown whether the inhibitors of GPX4 or FSP1 can safely induce ferroptosis *in vivo* without severe side effects. The genetic removal of GPX4 leads to acute renal injuries [88] and hepatocyte death that can be preventable by vitamin E [89]. Therefore, we would not be surprised if GPX4 inhibitors have significant liver and renal toxicities. In contrast, the genetic removal of FSP1 in mice results in modest phenotypes [90, 91]. Therefore, targeting FSP1 may have fewer side effects and better tolerated than GPX4 inhibitors.

We expect that ferroptosis will emerge as a promising therapy to enhance the efficacy of immunotherapy, chemotherapeutics, and PARP inhibitors for advanced OVCA. However, much work remains to be accomplished toward that goal. Especially, the identification of robust predictive biomarkers of ferroptosis sensitivity to select tumors that are most likely to respond. Additionally, it is critical to identify the best means of inducing *in vivo* ferroptosis as well as optimize the combination strategies. In the long-term, we expect that the similar targeting of the altered metabolisms in OVCA may present an entirely new avenue of therapeutic opportunity for OVCA, which can be incorporated with current treatments.
